# Acute Middle Gastrointestinal Bleeding Risk Associated with NSAIDs, Antithrombotic Drugs, and PPIs: A Multicenter Case-Control Study

**DOI:** 10.1371/journal.pone.0151332

**Published:** 2016-03-15

**Authors:** Naoyoshi Nagata, Ryota Niikura, Atsuo Yamada, Toshiyuki Sakurai, Takuro Shimbo, Yuka Kobayashi, Makoto Okamoto, Yuzo Mitsuno, Keiji Ogura, Yoshihiro Hirata, Kazuma Fujimoto, Junichi Akiyama, Naomi Uemura, Kazuhiko Koike

**Affiliations:** 1 Departments of Gastroenterology and Hepatology, National Center for Global Health and Medicine, Tokyo, Japan; 2 Department of Gastroenterology, Graduate School of Medicine, The University of Tokyo, Tokyo, Japan; 3 Ohta Nishinouchi Hospital, Fukushima, Japan; 4 Department of Gastroenterology, JR Tokyo General Hospital, Tokyo, Japan; 5 Department of Gastroenterology, Japanese Red Cross Medical Center, Tokyo, Japan; 6 Department of Gastroenterology, Tokyo Metropolitan Police Hospital, Tokyo, Japan; 7 Department of Internal Medicine and Gastrointestinal Endoscopy, Saga Medical School, Saga, Japan; 8 Department of Gastroenterology, Tokyo Metropolitan Police Hospital, Chiba, Japan; Mathematical Institute, HUNGARY

## Abstract

**Background:**

Middle gastrointestinal bleeding (MGIB) risk has not been fully investigated due to its extremely rare occurrence and the need for multiple endoscopies to exclude upper and lower gastrointestinal bleeding. This study investigated whether MGIB is associated with the use of non-steroidal anti-inflammatory drugs (NSAIDs), low-dose aspirin (LDA), thienopyridines, anticoagulants, and proton-pump inhibitors (PPIs), and whether PPI use affects the interactions between MGIB and antithrombotic drugs.

**Methods:**

In this multicenter, hospital-based, case-control study, 400 patients underwent upper and lower endoscopy, 80 had acute overt MGIB and 320 had no bleeding and were matched for age and sex as controls (1:4). MGIB was additionally evaluated by capsule and/or double-balloon endoscopy, after excluding upper and lower GI bleeding. Adjusted odds ratios (AOR) for MGIB risk were calculated using conditional logistic regression. To estimate the propensity score, we employed a logistic regression model for PPI use.

**Results:**

In patients with MGIB, mean hemoglobin level was 9.4 g/dL, and 28 patients (35%) received blood transfusions. Factors significantly associated with MGIB were chronic kidney disease (p<0.001), liver cirrhosis (p = 0.034), NSAIDs (p<0.001), thienopyridines (p<0.001), anticoagulants (p = 0.002), and PPIs (p<0.001). After adjusting for these factors, NSAIDs (AOR, 2.5; p = 0.018), thienopyridines (AOR, 3.2; p = 0.015), anticoagulants (AOR, 4.3; p = 0.028), and PPIs (AOR; 2.0; p = 0.021) were independently associated with MGIB. After adjusting for propensity score, the use of PPIs remained an independent risk factors for MGIB (AOR, 1.94; p = 0.034). No significant interactions were observed between PPIs and NSAIDs (AOR, 0.7; p = 0.637), LDA (AOR, 0.3; p = 0.112), thienopyridine (AOR, 0.7, p = 0.671), or anticoagulants (AOR, 0.5; p = 0.545).

**Conclusions:**

One-third of patients with acute small intestinal bleeding required blood transfusion. NSAIDs, thienopyridines, anticoagulants, and PPIs increased the risk of acute small intestinal bleeding. However, there were no significant interactions found between antithrombotic drugs and PPI use for bleeding risk.

## Introduction

Gastrointestinal (GI) bleeding is classified into the three categories of upper GI bleeding (UGIB), middle GI bleeding (MGIB), and lower GI bleeding (LGIB). When the source of GI bleeding is between the ampulla of Vater and the terminal ileum, it is defined as MGIB[1,2]. Acute overt MGIB is extremely rare; it has been reported to be l.2% among patients with acute GI bleeding[2]. Furthermore, the diagnosis of MGIB requires multiple endoscopies, such as capsule endoscopy or double-balloon endoscopy, besides upper and lower endoscopy to exclude UGIB and LGIB. Therefore, it seems unlikely that any single-center study would be able to recruit a sufficient number of patients with acute overt MGIB that had been diagnosed strictly.

Non-steroidal anti-inflammatory drugs (NSAIDs), low-dose aspirin (LDA), thienopyridines, and anticoagulants are known to induce UGIB[3] and LGIB[4,5]. Although these drugs have the potential to cause mucosal injury in the small intestine[6–11], their effect has not been fully investigated on the serious adverse GI event of acute overt MGIB.

Proton-pump inhibitors (PPIs) have been widely used for effective and preventive treatment of UGIB related to antithrombotic drugs[12]. However, a recent study suggested that PPIs increase the risk of small intestinal damage induced by LDA or NSAIDs [9–11]. Microbiota and cytokines play an important role in the pathogenesis of NSAID-induced enteropathy, and therefore acid suppression with PPI use may result in bacterial overgrowth, in turn leading to small bowel injury [13–15]. However, no data are currently available on the effect of PPIs on acute overt MGIB risk.

We previously conducted a case-control study on the effect of PPI use on acute UGIB and acute LGIB[16,17]. However, we could not evaluate MGIB risk because of the small number of cases. In the present study, we conducted a multicenter study to investigate MGIB, using both upper and lower endoscopy to exclude UGIB and LGIB, and assessed the use of antithrombotic drugs and PPIs on the day of endoscopy. Specifically, we sought to ascertain the effects of NSAIDs, LDA, thienopyridine, anticoagulants, and PPIs on MGIB and to determine whether PPIs affect the interactions between MGIB and antithrombotic drugs.

## Material and Methods

### Study design, setting, and participants

This retrospective, hospital-based, case-control study (case-control ratio, 1:4) was conducted between January 2009 and December 2014 at five hospitals (referral, territorial, or emergency) in the Metropolitan Tokyo area: Tokyo Metropolitan Police Hospital, The University of Tokyo Hospital, JR Tokyo General Hospital, the National Center for Global Health and Medicine, and the Japanese Red Cross Medical Center. Data were collected from a prospectively recorded electronic endoscopic database and questionnaires, and supplemented by medical chart reviews. The endoscopic database is a searchable collection of records that gastroenterologists prospectively input within 1 day of performing capsule endoscopy, esophagogastroduodenoscopy (EGD), or colonoscopy. These records consist of the gastroenterologists’ interpretation of endoscopic findings such as imaging findings, suspected diagnosis, and differential diagnosis.

This study was conducted according to the principles of the Declaration of Helsinki and was approved by the ethics committee of National Center for Global Health and Medicine (No. 814). The need for patient consent was waived because patient information was anonymized and deidentified before analysis.

### Cases

Cases were Japanese adult patients with acute overt MGIB who underwent at least three forms of endoscopy: EGD, colonoscopy, and capsule endoscopy. To diagnose acute overt MGIB, we applied the following strict diagnostic strategy[1,2,18]. First, from the electronic endoscopy database we selected 442 patients with suspected acute small bowel bleeding who underwent EGD, colonoscopy, and capsule endoscopy and then we reviewed the endoscopic findings and clinical data for all patients to confirm none had endoscopic-verified UGIB (from the esophagus to duodenum) or endoscopic-verified LGIB (from the colon to anorectum). Second, we selected 107 patients who satisfied the definition of overt GI bleeding, namely, bleeding within the past 3 days that might result in hemodynamic instability, anemia, and/or the need for blood transfusion[1]. Third, we excluded 27 patients who on capsule endoscopy and/or double-balloon endoscopy had an unclear lesion responsible for the bleeding such as petechiae/red spots (category 1) or small erosions (category 2 or 3), as previously described[18]. Finally, 80 endoscopically verified acute overt MGIB patients were selected for analysis.

### Controls

Controls were Japanese adult patients who were admitted to the hospital or were outpatients attending for cancer screening or other reasons (e.g., irritable bowel syndrome or functional dyspepsia) and had no signs of GI bleeding within the past 1 year based on endoscopy, clinical, and laboratory findings. During the study period, 1,913 patients underwent both EGD and colonoscopy and also completed, within 1 month of the procedures, a prospectively conducted questionnaire at NCGM that asked about lifestyles, co-morbidities, and medication use. The methodology for the questionnaire study has been reported elsewhere [4,16,17]. We then excluded patients whose use of medications was unknown (n = 13) and patients who had ulceration or a hemorrhagic lesion that involved the stomach, duodenum, or colon and rectum as detected by endoscopy (n = 231). This left 1,673 patients without any evidence of GI bleeding as controls who were matched for age (10-year age groups) and sex at a case-to-control ratio of 1:4.

### Capsule endoscopy strategy and treatment of MGIB

After 12 hours of fasting and oral administration of simethicone 40 mg, capsule endoscopies were performed using a PillCam® SB, or SB2 capsule endoscope (Covidien, Dublin, Ireland)[19]. The capsule endoscopy findings were prospectively recorded in the electronic database by expert gastroenterologists with 3 to 7 years of capsule endoscopy experience at each participating hospital. These findings were then re-evaluated by two expert researchers (N.R. and Y.A.) based on a standard classification[20] and a consensus was reached on the diagnosis. Double-balloon endoscopy was performed when a positive finding was noted on capsule endoscopy, particularly in patients with blood pooling in the small bowel that required further analysis, those with stigmata of recent hemorrhage that required further procedures, or those with a tumor-like lesion that required a biopsy. In cases where a bleeding lesion located in the terminal ileum or the lower bend of the duodenum was identified, we used colonoscopy or EGD instead of double-balloon endoscopy. After full bowel preparation with polyethylene glycol solution, double-balloon endoscopies were performed using an electronic video endoscope (Fujifilm Corporation, Tokyo, Japan). Endoscopic hemostatic intervention was then performed according to the following algorithm: 1) vascular lesions such as angioectasia and ulcerous lesions were treated by electronic coagulation, argon plasma coagulation, clipping, ligation, or an injection of polidocanol; 2) varices were treated by endoscopic band ligation; and 3) bleeding polyps were treated by endoscopic mucosal resection. In the case that endoscopy failed or patients had a tumor, we performed interventional radiology or surgery for GI bleeding. When hemoglobin levels fell below 7.0 g/dL or 8.0 g/dL in patients with unstable vital signs during hospitalization, a blood transfusion was indicated.

### Data sources and measurement

We collected clinical information from the abovementioned electronic database or by questionnaire. Patients were asked about their use of the following drugs, where drug use was defined as intermittent or regular oral administration within 1 month prior to the bleeding event: NSAIDs (loxoprofen, diclofenac, naproxen, etodolac, zaltoprofen, meloxicam, lornoxicam, or celecoxib), LDA (100 mg enteric-coated aspirin or 81 mg buffered aspirin), thienopyridines (ticlopidine or clopidogrel), anticoagulants (warfarin, dabigatran etexilate, apixaban, or rivaroxaban), and any dosage of PPI (omeprazole, esomeprazole, lansoprazole, or rabeprazole). Regular drug use was defined as a history of taking for > 1 month LDA, thienopyridines, anticoagulants, or PPIs.

We collected data on the co-morbidities of hypertension, diabetes mellitus, dyslipidemia, chronic kidney disease (CKD), liver cirrhosis, chronic obstructive pulmonary disease (COPD), connective tissue disease, and prior history of peptic ulcer disease, which has been associated with GI bleeding risk[21,22]. CKD was defined as receiving hemodialysis or peritoneal dialysis or a glomerular filtration rate <60 mL/min/1.73 m^2^ for 3 months.

### Statistical analysis

Characteristics were compared between MGIB and non-bleeding patients using Pearson’s chi-square test, Fisher’s exact test, or Mann–Whitney U test, as appropriate. Conditional logistic regression analysis was used to calculate the odds ratio (OR) and 95% confidence interval (CI) as an estimate of an MGIB event associated with drug exposure. Multivariate analysis included factors that were significantly associated with MGIB on univariate analysis (CKD, liver cirrhosis, NSAIDs, thienopyridines, anticoagulants, and PPIs), and possible risk factor (LDA use). To reduce the effect of selection bias and potential confounders for PPI use whenever possible, we applied the propensity score[23] to control for confounders in multivariate analysis in addition to the aforementioned factors. To estimate the propensity score, we employed a logistic regression model for PPI use including 9 factors that were shown to be different (p<0.10) between PPI use and non-use: being elderly (age ≥65 years), current drinker, current smoker, hypertension, dyslipidemia, chronic kidney disease, NSAIDs, thienopyridines, and anticoagulant use. Using a multivariate logistic model with adjustment for confounding factors, we further investigated interactions between PPI and NSAIDs, PPI and LDA, PPI and thienopyridine, and PPI and anticoagulants, as well as their effect on MGIB. Statistical significance was set at p < 0.05 and statistical analyses were carried out using Stata software, version 13 (StataCorp, College Station, TX).

## Results

Data was analyzed for 80 patients that experienced acute overt MGIB and 320 controls that had no GI bleeding during the study period. Patient characteristics are shown in Table 1.

**Table 1:** Patient characteristics (N = 400).

	Acute MGIB (n = 80)	Controls (n = 320)	P value
Age group (years)			
20–29	1 (1.3)	4 (1.3)	
30–39	5 (6.3)	20 (6.3)	
40–49	12 (15.0)	48 (15.0)	
50–59	7 (8.8)	28 (8.8)	
60–69	20 (25.0)	80 (25.0)	
70–79	19 (23.8)	76 (23.8)	
≥ 80	16 (20.0)	64 (20.0)	1.000
Male	44 (55.0)	176 (55.0)	1.000
Current drinker	36 (50.0)	151 (47.2)	0.666
Current smoker*	9 (12.3)	53 (16.6)	0.371
Co-morbidities			
Hypertension	9 (12.3)	53 (16.6)	0.371
Diabetes mellitus	6 (7.5)	45 (14.1)	0.115
Dyslipidemia	13 (16.3)	63 (19.7)	0.483
Chronic kidney disease	14 (17.5)	14 (4.4)	< 0.001
Liver cirrhosis	11 (13.8)	21 (6.6)	0.034
COPD	0	0	NA
Connective tissue disease	2 (2.5)	13 (4.1)	0.511
Prior peptic ulcer disease	12 (15)	54 (16.9)	0.686
Drugs			
NSAIDs	19 (23.8)	23 (7.2)	< 0.001
Low-dose aspirin	11 (13.8)	40 (12.5)	0.764
Thienopyridines	14 (17.5)	13 (4.1)	< 0.001
Clopidogrel	3 (3.8)	8 (2.5)	0.541
Ticlopidine	11 (13.8)	5 (1.6)	< 0.001
Anticoagulants	8 (10.0)	8 (2.5)	0.002
Warfarin	2 (2.5)	7 (2.2)	0.866
Other anticoagulants	6 (7.5)	1 (0.3)	< 0.001
PPIs	34 (42.5)	64 (20.0)	< 0.001
Omeprazole	12 (15.0)	20 (6.3)	0.001
Esomeprazole	2 (2.5)	2 (0.63)	0.132
Rabeprazole	6 (7.5)	11 (3.4)	0.107
Lansoprazole	14 (17.5)	32 (10.0)	0.060
Initial hemoglobin level (g/dL), median (range) [IQR]	9.5 (3.9–15.8) [7.7, 10.9]	NA	NA
Transfusion requirement	28 (35.0)	NA	NA
Units of transfusion per patient, median (range) [IQR]	0 (0–38) [0, 2]	NA	NA

Values in parentheses are percentages.

*Smoking data were collected from 73 MGIB patients and all 320 controls.

Abbreviations: COPD, chronic obstructive pulmonary disease; IQR, interquartile range; MGIB, middle gastrointestinal bleeding; NA, not applicable; NSAIDs, non-steroidal anti-inflammatory drugs; PPIs, proton-pump inhibitors

Factors significantly associated with MGIB were CKD, liver cirrhosis, NSAIDs, thienopyridines, anticoagulants, and PPIs. In patients with MGIB, median hemoglobin level was 9.5 g/dL at the time of diagnosis, and 28 patients (35%) received blood transfusions.

Bleeding source and therapy for acute MGIB are shown in Table 2. Of the 80 patients with MGIB, 59% had an ulcerous lesion, 51% underwent double-balloon endoscopy, and 24% received endoscopic therapy. After receiving a diagnosis of MGIB, 17.5% (14/80) of MGIB patients had rebleeding, with a mean follow-up of 365.6 days.

**Table 2:** Bleeding source and therapy for acute MGIB (n = 80).

Diagnosis	n (%)
Ulcerous lesion	47 (58.8)
Angioectasia/ angiodysplasia	15 (18.8)
Ulcerated or erosive tumor^†^	7 (8.8)
Bleeding from small bowel diverticula	4 (5.0)
Others^¶^	7 (8.8)
Therapy	
Double-balloon enteroscopy	41 (51.3)
Endoscopic therapy	19 (23.8)
Intervention radiology	2 (2.5)
Surgery	4 (5.0)

Values in parentheses are percentages.

^†^Tumors included gastrointestinal stromal tumor (n = 4), adenocarcinoma (n = 1), metastasis from lung cancer (n = 1), and malignant lymphoma (n = 1).

^¶^Others included small bowel varices (n = 4), and small intestinal polyps (n = 2), and slow active bleeding lesion (n = 1). There were no patients with Behçet's disease or Crohn's disease.

Abbreviations: MGIB, middle gastrointestinal bleeding.

Crude and adjusted ORs of MGIB associated with antithrombotic drug and PPI use are shown in Table 3. Multivariate analysis revealed that the use of NSAIDs, thienopyridines, anticoagulants, and PPIs were independent risk factors for MGIB. After adjusting for propensity score, the use of PPIs remained an independent risk factors for MGIB on multivariate analysis (adjusted OR, 1.94 [1.05–3.59]; p = 0.034).

**Table 3:** MGIB risk associated with use of antithrombotic drugs and PPIs and its interaction (n = 400).

	Crude OR	P	Adjusted OR^†^	P	Interaction with PPIs Adjusted OR^¶^	P
NSAIDs	3.7 (1.9–7.2)	< 0.001	2.5 (1.2–5.3)	0.018	0.3 (0.1–1.6)	0.178
Low-dose aspirin	1.1 (0.5–2.4)	0.756	0.9 (0.4–2.3)	0.907	0.3 (0.05–1.4)	0.122
Thienopyridines	5.0 (2.2–11.4)	< 0.001	3.2 (1.3–8.4)	0.015	1.4 (0.2–10.1)	0.723
Anticoagulants	4.7 (1.6–13.7)	0.005	4.3 (1.2–15.4)	0.028	1.0 (0.1–10.3)	0.977
PPIs	2.9 (1.7–4.8)	< 0.001	2.0 (1.1–3.6)	0.021	NA	NA

Values in parentheses means 95% confidential interval.

^†^Adjustment for the use of chronic kidney disease, liver cirrhosis, NSAIDs, low-dose aspirin, thienopyridine, anticoagulants, and PPIs.

^¶^Adjustment for the use of chronic kidney disease, liver cirrhosis, NSAIDs, low-dose aspirin, thienopyridine, and anticoagulants.

Abbreviations: MGIB, middle gastrointestinal bleeding; NA, not applicable; NSAIDs, non-steroidal anti-inflammatory drugs; PPIs, proton-pump inhibitors.

No significant interactions were observed in the interaction model (Table 3) between PPIs and NSAIDs (p = 0.178), LDA (p = 0.122), thienopyridines (p = 0.723), or anticoagulants (p = 0.977), between NSAIDs and LDA (adjusted OR, 0.77 [0.082–7.31]; p = 0.822), or between LDA and thienopyridines (adjusted OR, 0.16 [0.018–1.42]; p = 0.100).

## Discussion

We explored the association of antithrombotic drug or PPI use with the risk of acute-overt MGIB in patients who underwent both EGD and colonoscopy and age- and sex-matched controls. First, we found that one-third of patients with acute MGIB required blood transfusion. Second, we found that NSAID, thienopyridine, anticoagulants, and PPI use were independent risk factors for MGIB. Third, there were no significant interactions seen between PPIs and NSAIDs, LDA, thienopyridines, or anticoagulants for MGIB. As far as we know, this is the first report of PPI use as an independent risk factor for acute-overt MGIB.

In an animal study, rats treated with omeprazole had more intestinal enteric bacteria than controls, and the reduction of gastric injury by omeprazole was accompanied by clear exacerbation of small intestinal ulceration and bleeding[13]. In clinical studies, PPI combined with NSAID or LDA use was reported to increase the risk of small mucosal injury compared with NSAIDs or LDA alone[9–11]. PPI use was also found to be an independent risk factor for multiple small bowel erosions/ulcers and decreased hemoglobin levels in 113 chronic NSAID users examined using capsule endoscopy[24]. PPI therapy is effective for the prevention of UGIB[12,16], and PPI use did not lead to an increased or decreased risk of LGIB[17]. On the other hand, our data and those of previous studies [9,10] indicate that PPI use has the potential to increase the risk of MGIB. Therefore, the recurrence rate might be different between UGIB, MGIB, and LGIB especially when we prescribe PPIs at the time of discharge after acute GI bleeding. To date, several clinical studies have shown that mucoprotective drugs such as misoprostol and rebamipide have a preventive effect in NSAID-induced small intestinal injuries[25]. Therefore, to prevent rebleeding related to NSAID or LDA use, administration of a PPI together with a mucoprotective drug may be needed at discharge especially when there is the possibility of MGIB.

In agreement with earlier studies, this study has shown a significant association between NSAID use and overt MGIB. However, outcomes in previous studies were not overt bleeding, but endoscopy-proven small bowel injury (a surrogate marker). Among 40 healthy volunteers after 2 weeks of diclofenac ingestion, capsule endoscopy showed the presence of a new lesion in 27 subjects (68%) [6]. Mucosal breaks were found in 44 of 120 (28%) NSAID users but none were seen in 60 healthy controls[7]. Despite the fact that the design and outcome of these previous studies were different from the present one, their results support ours.

In the present study, however, LDA use was not a risk factor for MGIB. It remains unclear whether LDA is really harmful to the small bowel. In a study of 20 healthy subjects after 2 weeks of LDA ingestion, capsule endoscopy detected 10 (50%) with mucosal damage, but only 1 had large erosions/ulcers[8]. In clinical practice, LDA is usually indicated for long-term use, so a conclusion that LDA causes small intestinal ulcer cannot be drawn from these short-term studies. Furthermore, previous studies have suggested a lower risk of small bowel injury in LDA users than in NSAID users[26,27].Therefore, we suggest that LDA has the potential to cause small bowel mucosal injury[8], but not enough to cause bleeding, compared with non-LDA NSAIDs.

We found that thienopyridine use was an independent risk factor for MGIB. Similarly, in a previous study of OGIB patients who were taking antithrombotic drugs, capsule endoscopy most frequently detected ulcers in those taking LDA and thienopyridine compared with those taking LDA, thienopyridine, or warfarin alone[28]. However, because only scant data on the association between thienopyridine use and small intestinal injury are available, a further cohort or case-control study is needed to confirm this.

In our interaction model, we found that PPI did not affect the risk of MGIB in NSAID or LDA users. Endo et al. prospectively evaluated the small bowel using capsule endoscopy in LDA users and showed that PPI use was an independent risk factor for mild mucosal breaks[9]. These conflicting findings may reflect different outcomes for mucosal changes and overt bleeding.

Our study has some limitations. First, this was a retrospective study, and selection bias was potentially present because, for example, endoscopy could not be performed for critically ill or intubated patients. Second, we could not collect sufficient information on the frequency, dose, or duration of drug intake, particularly for NSAIDs. Third, it is possible that some patients had concurrent bleeding lesions such as UGIB and MGIB or LGIB and MGIB lesions. One study showed that the location of bleeding intestinal angioectasias in patients who had undergone complete endoscopy were the stomach and jejunum (17%) and the jejunum and right colon (14.3%)[29]. Despite the fact that performing EGD, colonoscopy, and double-balloon endoscopy concurrently for all patients with acute GI bleeding is challenging in clinical practice, inclusion bias might have existed in our study and further studies may be needed to elucidate the association between PPIs and concurrent MGIB and other GI bleeding sources. Fourth, although we conducted an age- and sex-matched case-control study and applied the propensity score for PPI use which was used to control for confounders in multivariate analysis, unmeasurable confounders (i.e., indication, underlying disease, medication, and lifestyle factors) associated with PPI use may exist.

Despite these limitations, there are several important strengths of this study. First, acute MGIB is reported to be extremely rare among the types of acute GI bleeding, but the number of MGIB cases was relatively large (n = 80) in our present multicenter study. Second, all subjects underwent EGD and colonoscopy, including control subjects, and strict criteria were applied to diagnose MGIB cases[1,2,18]. Third, control subjects had been matched for age and sex (1:4 case-to-control ratio), which increases the precision and power of case-control studies[30].

In conclusion, use of NSAIDs, thienopyridine, and PPIs significantly increased the risk of acute-overt MGIB. However, no significant interactions were seen between antithrombotic drugs and PPI use for MGIB risk.
